# Three-dimensional space use during the bottom phase of southern elephant seal dives

**DOI:** 10.1186/s40462-017-0108-y

**Published:** 2017-08-31

**Authors:** Yves Le Bras, Joffrey Jouma’a, Christophe Guinet

**Affiliations:** Centre d’Études Biologiques de Chizé, UMR 7372, CNRS-ULR, Villiers-en-bois, 79360 France

**Keywords:** Diving behaviour, Three-dimensional path reconstruction, Prey density, Prey distribution, Prey patch

## Abstract

**Background:**

In marine pelagic ecosystems, the spatial distribution of biomass is heterogeneous and dynamic. At large scales, physical processes are the main driving forces of biomass distribution. At fine scales, both biotic and abiotic parameters are likely to be key determinants in the horizontal and vertical distribution of biomass, with direct consequences on the foraging behaviour of diving predators. However, fine scale three-dimensional (3D) spatial interactions between diving predators and their prey are still poorly known.

**Results:**

We reconstructed and examined the patterns of southern elephant seals 3D path during the bottom phase of their dives, and related them to estimated prey encounter density. We found that southern elephant seal tracks at bottom are strongly dominated by a single horizontal direction. In high prey density areas, seals travelled shorter distances but their track remained strongly orientated according to a main linear direction. Horizontal, and more importantly, vertical deviations from this main direction, were related negatively to the estimated prey density. We found that prey encounter density decreased with diving depth but tended to be more predictable.

**Conclusion:**

Southern elephant seal behaviour during the bottom phase of their dives suggest that the prey are dispersed and distributed into layers in which their density relates to the vertical spread of the layer. The linear trajectories performed by the elephant seals would allow to explore the largest volume of water, maximizing the opportunities of prey encounter, while travelling great horizontal distances.

**Electronic supplementary material:**

The online version of this article (doi:10.1186/s40462-017-0108-y) contains supplementary material, which is available to authorized users.

## Background

Distributions of predators and prey are necessarily linked. Optimal foraging theory [1–3] predicts that a predator should seek out areas with high prey density while prey should avoid high predator density areas [4]. The correlation between the spatial distributions of predator and prey depends on the balance between the responses of one to another [5]. In the case of a mobile predator that feeds on a more static prey, the spatial distributions of the predator and of the prey are expected to be positively correlated [5]. For instance, diel vertical migrations performed by myctophids [6] are related to a similar pattern in the diving depth of elephant seals, *Mirounga angustirostris* [7] and *M. lenonina* [8–10]. Consequently, the hypothesis that movements of predators mimic the spatial patterns of their prey is commonly encountered in the bird and marine mammal literature. A typical example is the detection of Area-restricted search behaviour [11, 12] (ARS) from GPS tracks to infer the location and characteristics of important feeding areas of various marine predators [13–18].

Studying the distributions of predators and prey as well as their interactions is particularly challenging in the open ocean, because of the dynamic nature of this environment and the difficulty of observing the animals. In the last few decades, technological advances have driven the emergence of bio-logging as a way to simultaneously monitor the activity of free-ranging marine predators and sample their physical environment. The use of electronic devices embedded on free-ranging animals have provided novel insights into the foraging behaviour and habitat of marine predators at large and intermediate scales. For example, it has been highlighted that large and meso scale oceanographic structures such as fronts, eddies and filaments are of significant importance to the foraging ecology of top predators [19–25]. At fine scales, both biotic and abiotic parameters are likely to be key determinants in the dynamics of biomass distribution [26, 27], but these processes are still poorly understood. To study fine scale patterns in prey distribution, and their mechanistic relationships with predator behaviours, we used bio-logging data collected by southern elephant seals (SES) and focused our interest on the scale of a dive bottom phase. Indeed, this dive phase represents a fundamental organizational unit of the foraging strategy for many diving predators, including SES, where most of feeding occurs [28–34].

Southern elephant seals can dive at an average depth of 400 m (up to 2000 m, [35]) and explore a large extent of the water column. Their foraging strategy can be modified by adjusting both horizontal and vertical movements [31, 36, 37], therefore it is worth examining how the three spatial dimensions are involved in the interactions between diving predators and their prey. However, most studies investigating the space use by marine predators have either analysed animals’ behaviour from their GPS track (time + 2D approach), or from time-depth data (time + 1D approach). Some efforts have been made to combine these two approaches – horizontal dimensions at surface and time-depth dive profiles – in order to examine the foraging strategies according to horizontal and vertical dimensions (time + pseudo 3D approach) [38–40]. However, a detailed understanding of how diving predators use their 3D spatial environment and interact with prey requires the actual reconstruction of their 3D path underwater. This is nowadays achievable using bio-logging data from large diving predators that are able to carry sophisticated loggers with minimal disturbance [36, 41–45]. Such loggers can also provide information regarding the likely occurences of prey encounters [46–48]. Three-dimensional path analysis has started to provide new insights into the behaviour of elephant seals [42, 43] and other diving predators [36, 49–53], but also into the fine scale patterns of their prey distribution [45].

In this study, six datasets with acoustic recording, tri-axial acceleration and magnetometry, sampled at high frequency, allowed us to reconstruct the three-dimensional underwater path of SES using well established dead-reckoning methods [54]. According to the optimal foraging theory, the predators should exhibit the greatest residency time in the highest prey density grounds, but the general shape of the animal path can also convey information on the predator-prey interactions. Complementarily to ARS-like approaches that focus on specific part of the trajectories with high residency time, we decided to extract the main trends of the SES path in dives’ bottom phases. We described the 3D space use exhibited by SES at the bottom phase of their dives using principal component analysis, and assessed the volumetric density of prey encounter events under various prey detection range scenarios. Finally, to explain how the SES space use could relate to its perception range and to the fine-scale patterns in prey distribution, we examined relationships between the estimated prey encounter densities and the three-dimensional diving behaviour in bottom phase trajectories.

## Methods

### Deployment of devices and data collection

During the breeding seasons (October and November) of 2011 and 2012, a total of six SES females of the Kerguelen Islands (49 °21^′^0^′′^ S, 70 °13^′^0^′′^ E), were equipped with an acoustic tag (Acousondes^TM^ model 3A, manufactured by Acoustimetrics, Greeneridge Sciences, Inc, USA) and a Time-Temperature-Depth Fastloc GPS data logger (SPLASH10-F^TM^ manufactured by Wildlife Computers, USA) to collect locations while the animals were at sea. The tags were programmed to sample depth, light and temperature at 1 Hz, tri-axial (longitudinal, lateral and vertical axes of the logger) body acceleration, tri-axial earth magnetic field at 5 Hz and sound. Animals were captured with a canvas head-bag and anaesthetized using a 1:1 combination of Tiletamine and Zolazepam (Zoletil 100) injected intravenously [55]. Using quick-setting Araldite (Araldite AW 2101), the tags were glued on the seals (acoustic tags on their back, GPS tags on their head) so that longitudinal axes of the animals and loggers aligned. Details about the length and weight of the equipped animals are provided in Table 1 (average lengths of 2.37±*S* *D*=0.12 m and average weights of 277.67±*S* *D*=47.31 kg). Passive acoustic recording is power-consuming. To extend the acoustic sampling on longer periods, we programmed the tags to record sound at a frequency of 6 kHz for three hours every 12 h in 2011 and at a frequency of 12.2 kHz for three hours every 24 h for the four individuals equipped in 2012.

**Table 1:** Deployment details. All individuals are post-breeding females

SES name	Length (m)	Weight (kg)	n 3D dives	/	n dives	Recording
						duration (day)
2011-16	2.54	255	144	/	822	13
2011-18	2.28	245	238	/	1081	13
2012-01	2.32	230	248	/	1945	24
2012-02	2.35	362	68	/	409	12
2012-04	2.48	282	50	/	289	4
2012-08	2.25	292	244	/	1777	29

### Dive analysis

Unless otherwise specified, data processing and analysis described in this section were performed using the R statistical software [56]. The custom code used for the archive data processing is available online as a R package called rbl [57].

#### Dives and dive phases

We defined dives as periods where animals were continuously deeper than 15 m under the surface. This conservative threshold avoids considering brief sub-surface excursions as actual dives. Because there is a drift in the pressure readings of the tags over time, a zero offset correction of depth time sequence was applied prior to the delimitation of dives. Each dive was then divided into three phases – descent, bottom and ascent – using the method described in [58]. The bottom phase is defined as the period of a dive where the vertical speed signal, modelled using a polynomial of degree 4, stays under a threshold of 0.75 m s ^−1^. Modeling the vertical speed signal using a polynomial fit allows the method to be sensitive to the overall shape of the time-depth trajectory but not to small scale anomalies such as steps performed in the middle of the descent and ascent phases. The fourth degrees provide enough freedom for the model to handle V-shaped and squared (U-shaped) dives. Eventually, the vertical speed threshold was chosen after a blind experiment minimizing the difference between the automatic and the visual delineation of the bottom phases.

Drift dives are specific dives where SES are resting and/or digesting [59] and, as a result, not expected to react when encountering prey. Since our focus is on spatial patterns related to predator-prey interactions, we identified and removed these dives from our dataset prior to the statistical analysis.

#### Prey encounter events

To identify a prey encounter event we implemented the method described in [25, 58, 60] (but see [60] for details) on the acceleration data collected by the tags. The dynamic acceleration resulting from rapid movements was extracted from the three axes with an order 3 high-pass digital Butterworth filter with a normalized cut-off frequency of 2.4 Hz (performed with butter and filtflit functions from the signal package [61]). For each axis, a one-second fixed window was used to calculate the standard deviation every second. Signals were then processed using a moving standard deviation with a window size of five seconds. Finally, a two-mean clustering was performed for each signal to distinguish “high state” from “low state”. These successive operations are performed using the prey_catch_attempts function from the rbl package. A prey encounter event (hereafter PEE) is believed to be occurring when the three axes are simultaneously in “high state”. A continuous succession of “high state” is considered as a single PEE. A comparison of PEE detection results of this method derived from both the head-mounted and back-mounted accelerometer data is provided in [62, Additional file 2].

We counted PEE to obtain an indication of the number of prey encounters in the bottom phases but the corresponding prey types are not known.

### Three-dimensional path reconstruction

Three-dimensional reconstruction by dead-reckoning (also called “path integration”) is calculated by summing the successive velocity-vectors of the animal (in our case, every second) starting from a known location (in our case, a GPS location collected in the surface period preceding the dive). When the arrival point is known, the reconstructed track can be scaled to match the observed locations at departure and arrival (GPS location collected in the surface period following the dive) and reduce positional uncertainty [54].

Pitch, roll and heading angles describe the body posture of SES with respect to the direction of the earth’s gravity vector (pitch and roll angles) and earth’s magnetic vector (heading angle). Assuming that the animal always moves in the direction of their longitudinal axis, pitch and heading angles provide all the necessary directional information for 3D path reconstruction. The static acceleration is the gravity based acceleration component. It can be obtained by applying the appropriate low-frequency filter to the acceleration signal. As in [63], we used an order 3 low-pass digital Butterworth filter with a normalized cut-off frequency of 0.20 Hz applied to the three axes. The direction of the gravity vector according to the accelerometer provides a reference to calculate the pitch and roll angles of the SES (we used the pitch and roll functions from the animalTrack package [64]). The low-pass filter was applied to the magnetic data as well. The resulting signal, combined with pitch and roll information allows to calculate the heading angle (performed using tilt_compensate function from the animalTrack package).

Aside from body posture angles, 3D path reconstruction requires knowledge of the SES swimming speed. We assessed swimming speed of SES relatively to surrounding water using sound [65] recorded by acoustic tag. This task was performed in MATLAB using custom code which is available on request. We estimated the swimming speed (*v*_*seal*_) in descent and ascent phases from pressure changes (*v*_*z*_) and pitch angle (*α*): *v*_*seal*_=*v*_*z*_/*s* *i* *n*(*α*). Water flow noise level was calculated from the low-frequency noise extracted with a 110 Hz low-pass filter applied to acoustic data. Then, we calibrated the relationship between the water flow noise level and the swimming speed estimations and extrapolated it to predict swimming speed over the entire dive periods from noise level.

**Fig. 1:**
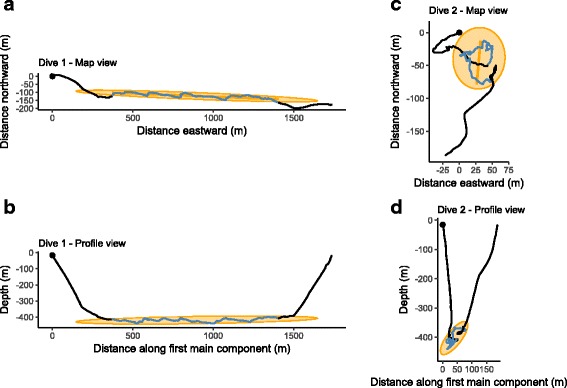
Two 3D dives examples. These dives have about the same depth but show two contrasting situations: on the left (**a** and **b**) the first main component explains a very large part of the total dispersion while on the right (**c** and **d**), the dispersion explained by the first main component is particularly low. The blue part represents the dive’s bottom phase. The black points give location of starting points. The orange ellipses stand for the 2D projections of a 3D ellipsoid whose axis are the three main components. On the “Map views” (**a** and **c**) the orange line display the direction of the first main component which is used to construct the best “Profile Views” (**b** and **d**). More example, in interactive 3D plots, may be found at https://github.com/SESman/SES_3Ddives

The 3D path (Fig. 1) of each animal was calculated by dead-reckoning (dead_reckoning function of the animalTrack package [64] applied to body posture angles, swimming speed and GPS data). The observed GPS location during the surface period preceding the dives is used as the starting point of the reconstructed path. Finally, the reconstructed paths are corrected so that their arrival point matches the observed GPS location collected during the surface period following the dives. This correction applies uniformly over the entire dive, simulating the effect of a current of constant speed and direction that would result in the difference observed between the reconstructed and GPS arrival points. Because the acoustic tags were programmed to record acoustic data only at specific hours of the day, we could not predict the swimming speed at night, and as a consequence, all the 3D dives of our dataset occurred during daytime.

### Shape of bottom phase trajectories

To describe how the SES used the 3D space during the bottom of their dives, we extracted the main components of the animals path during the bottom phases. This was achieved by implementing a Singular values Decomposition (calculation of the eigenvalues and eigenvectors) of the variance-covariance matrix of SES locations during dives’ bottom phase. The variance-covariance matrix can be seen as a linear application that would transform Gaussian noise (in 3D, this would be a spherical cloud of data points with maximum density at its center) into the observed data. Such a transformation can be decomposed into simpler transformations, rotations whose characteristics are described by the eigenvectors, and scaling described by the square roots of the eigenvalues. In our case study which relates to the extraction of main components of the 3D SES path, eigenvectors provides the direction of the main components and their eigenvalues quantifies how much of the dispersion (also called inertia) of SES locations each of these components can account for. All-equal eigenvalues would indicate that SES did not favour any direction of movement in their trajectories. The sum of the eigenvalues represents the total dispersion of the data. To describe the shape of the SES trajectories, we used the raw SES location data as well as eigenvectors and eigenvalues describing the main linear trends in the SES path. The variables that we calculated are listed and briefly described in Table 2. Additional explanations for the variables that require it is provided in the next paragraphs:

**Table 2:** Variables used to describe the shape of SES trajectories

Variable name	Brief description
Mean depth	Average depth in the bottom phase.
Total dispersion	Sum of the eigenvalues: *λ*_1_+*λ*_2_+*λ*_3_
First main component dispersion	Defined as $\frac {\lambda _{1}}{\lambda _{1} + \lambda _{2} + \lambda _{3}}$
Vertical and horizontal extent of first main component	These two variables describe the extent of SES exploration along the path of the first main component. See details in text.
Vertical and horizontal width	These variables quantify the vertical and horizontal spread of the deviations from the first main component path. See details in text.
Swimming speed variability	Standard deviation of SES swimming
	speed. See details in text.

**First main component dispersion** The first main component (abbreviated MC1) is the main component with the greatest eigenvalue, that is the primary direction of movement. A perfect balance between the directions of movements is characterised by a value of one third (in which case all eigenvalues are equal). Values larger than one third indicate that some directions of movement dominate in the SES path. The larger is this value the stronger is the dominance but a value of 1 corresponds to a perfectly linear path.

The first main component dispersion was fairly large while the first main component orientation revealed a strong consistency across dives (see results section). Conversely, the second and third main components far less dispersion and their orientation varied importantly from a dive to another. On this basis, we chose to describe the dispersion not explained by the first main component with behaviour metrics based on the horizontal and vertical dimensions rather than according to the second and third main components. Using this frame of reference which is both consistent between dives and meaningful in terms of biological and physical processes allows to simplify the interpretation of the models’ results.

**Vertical and horizontal extent of first main component** We calculated the horizontal and vertical distances separating the ends of the first main component. For robustness, we defined the ends as 10% and 90% quantiles of first main component scores instead of the minimum and maximum values.

**Fig. 2:**
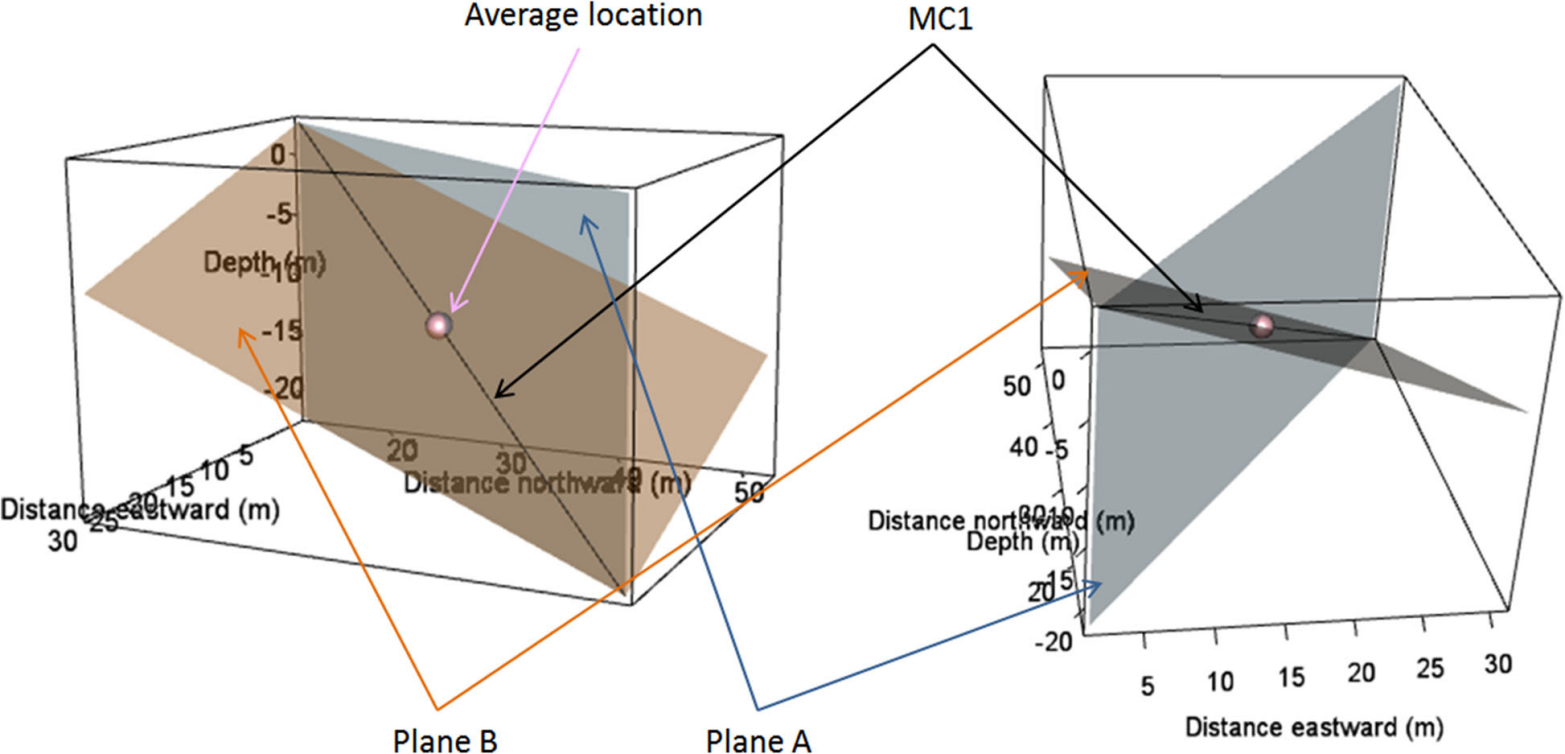
Schematic presenting the two reference planes for the calculation of vertical and horizontal width. The first main component is represented by the thick black line at the center. The blue (respectively orange) plane stands for plane A (respectively plane B). The pink sphere indicates the average location of the animal

**Vertical and horizontal width** To calculate these variables we defined two orthogonal planes: Plane A, passing through the gravity center of the trajectory and encompassing the first eigenvector and the gravity vector (blue plane on Fig. 2); Plane B, passing through the gravity center of the trajectory, encompassing the first eigenvector and a vector orthogonal to plane A (orange plane on Fig. 2). Horizontal width was defined as the range from the 10% to the 90% quantile of distances between SES locations and plane A. Vertical width was defined as the range from the 10% to the 90% quantile of distances between SES locations and plane B.

**Swimming speed variability** A high swimming speed variability is believed to be related to prey chasing [66], while a low swimming speed variability can indicate a drifting or gliding behaviour [67] which is believed to be related to low foraging effort ([59, 68]). Besides the shape-description variables, we used the standard deviation of the swimming speed during the bottom phase as a proxy of foraging effort in order to be able to distinguish between these behaviours.

### Volume of water prospected by SES at the bottom of their dives

We estimated the volume of water prospected by SES, that is to say the volume of water where SES would have been able to detect a prey during the bottom phase of a dive. To achieve this, we assumed that SES could see in any direction around their current position within a given radius. We considered three “detection-distance” scenarios for the sphere radius: a short distance of 1.5 m to simulate the case where prey catches would be opportunistic events as well as 9 and 18 m according to the foraging scale highlighted by [45] on the northern elephant seals. SES may exhibit an infinite variety of 3D path during their dives’ bottom phase, including turning back to visit the same areas several times. Hence, we could not use a generic equation to assess the volume of water they may have prospected. Instead, we used a numerical method called Monte Carlo integration. Details about the implementation and accuracy of this method are provided in Additional file 1. Once the prospected water volume was computed, we used it to compute a proxy of the prey encounter density at the bottom of SES dives, defined as the ratio between the number of PEE and the prospected water volume (expressed in *μ*PEEm^−3^ units).

### Statistical analysis

We modelled the prey density proxy (*μ*PEE per m^3^ of water prospected at bottom) according the descriptors of bottom trajectory previously described (mean depth, total dispersion, first main component dispersion, first main component extent on the horizontal and vertical dimensions, horizontal and vertical widths and standard deviation of the swimming speed) using Generalized Linear Models (GLM). We fitted three models, one for each detection radius.

We started the model selection with Poisson family GLMs, adapted to predict a count variable such as PEE count at bottom. The link function was set to logarithm (the standard link function for these GLMs) and the log-transformed volume of prospected water was included in the model as an offset term. With this implementation, we could model the PEE per unit of water prospected at bottom as response variable while using the appropriate count family distribution to predict the number of PEE at bottom.

These Poisson models indicated over-dispersion (*θ*=*σ* ^2^/*μ*, *θ*_1.5*m*_=2.93, *θ*_9*m*_=2.68, *θ*_18*m*_=2.73) so we switched to the more flexible Negative Binomial distribution (MASS package [69]) which allows for higher variance/mean ratio. We observed a large proportion of zeros in our data (27.44%) incorrectly predicted by the GLMs. We then tested the zero-inflated variants of Poisson and Negative Binomial models (using zeroinfl from the pscl package [70]). These models led to very significant improvement of Akaïke Information Criterion (AIC) and Vuong’s test (*p*-values were <0.01 for all models).

The explanatory variables considered for selection in the count part of the zero-inflated model were identical to the Poisson and Negative Binomial models. The choice of explanatory variable for the zero excess part was restricted to the non shape-description variables, individual identity, mean depth, swimming speed variability and log-transformed bottom duration (the latter was not included in the count part of the model). Seals’ identity captures variability due to differences in accelerometer attachment and individuals’ foraging behaviour. Swimming speed variability can account for the shift between drifting/gliding and active swimming behaviour in terms of foraging effort. Mean depth provides basic information about the environment which is a likely source of excess zeros. Finally, log-transformed bottom duration has an obvious link to the probability of PEE occurrence.

We tested quadratic effects for the swimming speed variability (in both count and zeros excess parts) and the vertical and horizontal extent of the trajectory across the first main component. We tested all the possible combinations of explanatory variables and ranked the best candidates according to AIC. For each radius, we selected the model with the best AIC where all explanatory variables were significant at level *α*=5*%*.

## Results

### Shape of SES trajectories at the bottom of their dives

**Fig. 3:**
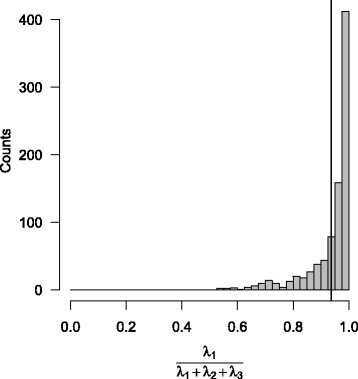
Contribution of the first eigenvalue to the total dispersion. A linear path would be indicated by a 100% contribution. Conversely, a well balanced 3D path would be indicated by equivalent contribution of the three eigenvalues to the total dispersion, that is a 33% contribution of the first eigenvalue. The black vertical line stands for the average, at 93.75% of the total dispersion

**Fig. 4:**
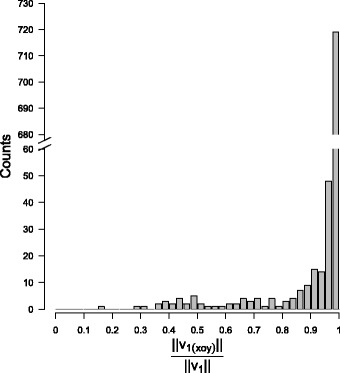
Length ratio between the first eigenvector projected on the horizontal plane (*v*_1(*xoy*)_) and the first eigenvector in 3D space (*v*_1_). A ratio of 0 indicates that the first main component is perfectly vertical while a ratio of 1 indicate that the first main component is perfectly horizontal

The first main component explained 93.75% ± *S* *D*=8.34% of the total dispersion (Fig. 3, Table 3). Moreover, the first main component was almost exclusively oriented in a horizontal direction (Fig. 4). The left dive on Fig. 1 is an example of a typical dive that exhibiting these characteristics. No bi-modality pattern is noticeable of Fig. 4 but, apart the obvious peak near one, a wide range of values is covered by a few observations. The vertical component of the first main component range between 17 to 76% in a few bottom phases (5%) which correspond to deep diving depths (680 m vs. 481 m, t-test *p*-value = 1.395×10^−13^). On average, SES travelled horizontal distances of 429m±*C* *V*=92*%* in their descent phases, 706m±*C* *V*=77*%* in bottom phases and 393m±*C* *V*=90*%* in ascent phases. Because the first main component are horizontally oriented, the first main component dispersion is highly correlated to the length of first main component according to the horizontal plane (first main component horizontal extent). As a result, when checking for multicollinearity before model selection, the first main component horizontal extent was removed.

**Table 3:** Descriptive statistics of the shape parameters of bottom trajectories

	Nb prey encounter event at bottom	Bottom duration (s)	Mean bottom depth (m)	Horizontal width (m)
Min	0.00	54.00	53.59	0.35
Max	28.00	1732.00	847.91	139.37
Median	2.00	451.00	435.78	20.39
Mean	3.37	500.39	441.86	23.20
SE mean	0.13	6.67	5.25	0.45
*C* *I*_95*%*_ mean	0.25	13.10	10.30	0.88
Variance	13.79	38572.57	23833.89	175.57
SD	3.71	196.40	154.38	13.25
	MC1 vertical width (m)	MC1 horiz. width (m)	Total dispersion (m^2^)	MC1 dispersion (%)
Min	0.04	36.80	562.55	53.30
Max	383.55	1775.42	401082.90	99.97
Median	26.80	409.09	24073.35	97.20
Mean	48.68	431.17	32638.69	93.75
SE mean	2.16	7.25	1110.50	0.28
*C* *I*_95*%*_ mean	4.23	14.23	2179.59	0.56
Variance	4023.07	45544.02	1067959847.62	69.62
SD	63.43	213.41	32679.65	8.34
	Vertical width (m)	Bottom speed SD (m s ^−1^)	Bottom vertical speed SD (m s ^−1^)	
Min	2.10	0.11	0.16	
Max	154.18	1.15	2.01	
Median	23.21	0.32	0.79	
Mean	31.45	0.35	0.85	
SE mean	0.80	0.01	0.01	
*C* *I*_95*%*_ mean	1.57	0.01	0.02	
Variance	556.80	0.03	0.09	
SD	23.60	0.17	0.30	

*n*=866 3D dives. Refer to methods for detailed explanation about what these variables represent and how they were computed

### Prey field density at the bottom of SES dives

The prey encounter density spread in a wide range according to the detection radius (two orders of magnitude, Table 4), due to the strong impact of this parameter on the estimates of the water volume prospected by SES. We can adopt the predator’s point of view by taking the inverse of the prey density estimates reported in the Table 4: considering prey detection radius of 1.5, 9 and 18 m, SES have to explore average volumes of 1.40×10^3^ m^3^, 5.39×10^4^ m^3^ and 2.08×10^5^ m^3^ respectively to encounter a prey during their dives’ bottom phase.

**Table 4:** Estimated prey encounter event density in dives’ bottom phases

Radius	Mean prey encounter event density ±*C* *I*_95*%*_			SD
1.5 m	715.09	±	53.70	760.13
9 m	18.56	±	1.45	20.58
18 m	4.81	±	0.39	5.45

*n*=866. Values expressed in *μ*PEE m^−3^ unless otherwise specified

### Prey density model

**Fig. 5:**
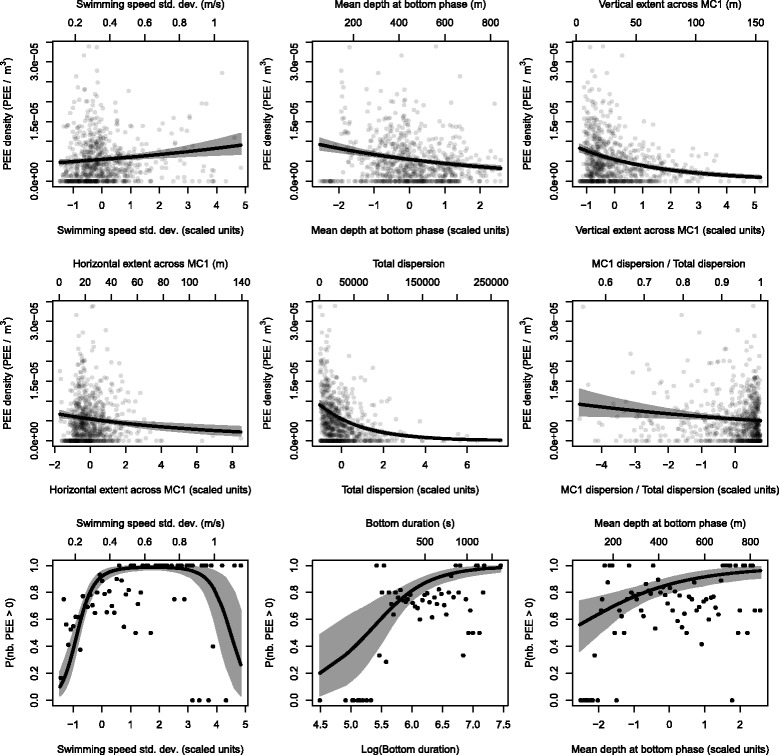
Estimated relationships between the PEE density proxy and the descriptive parameters of the bottom phase trajectories. Results obtained for 18 m radius. The top six graphics present the estimated effect of the count model and the bottom three graphics the estimated effects of the zero excess model. The thick black curves display the expected means at population level and the grey shades surrounding them stand for the 95% confidence interval of this expected mean. The figures obtained for 1.5 and 9 m radii are included in the Additional file 2

Results were consistent across all models with similar coefficient estimates and standard errors (Table 5). In the count part of the model, the strongest effect is observed for total dispersion for which a one SD increase is associated with 38% lower PEE density (Table 5). In decreasing order of effect strength, vertical width, mean bottom phase depth and horizontal width have negative effects in all three count models (Table 5). However, mean depth of bottom phase has a positive influence on the probability of catching at least one prey item (Table 5, Fig. 5). As expected, swimming speed variability had positive effects in both count and zero-excess parts of the model. Nonetheless we observed a negative quadratic effect for high values (>0.90m s^−1^) in the zero-excess part (Table 5, Fig. 5).

**Table 5:** Results of the three zero-inflated models

	

Please notice that, for historical reasons, the binomial part of zero-inflated models (labelled “Zero-excess”) predicts the absence of prey encounter event instead of presence. For better readability, the intercept estimates were not included in this table

Differences between the *r*=18 m and the two other models (*r*=9 and 1.5 m) were only observed for the least significant variable (Table 5). With *r*=18 m dispersion explained by first main component was selected whereas models with *r*<18 m favoured vertical extent of the first main component. These variables were associated with smaller PEE density for all models (Table 5). Partial regression lines for models with *r*<18 m are provided in the Additional file 2.

The dispersion parameter (*θ*) decreased with the chosen detection radius (*θ*_1.5*m*_=3.38, *θ*_9*m*_=3.24, *θ*_18*m*_=3.18). The final models could explain 17.04, 19.52 and 20.46% of the deviance of null models for 1.5, 9 and 18 m radius. Goodness of fit as indicated by the pseudo- *r* ^2^ (squared correlation coefficient between observed and predicted values of the PEE density) also increased with the detection radius (27.53%, 31.03%, 33.91% for 1.5, 9 and 18 m radius).

## Discussion

### Schooling behaviour

The principal component analysis revealed that SES trajectories at the bottom of their dives are strongly dominated by a path in a single direction (Fig. 3) as it was noted by [41] on northern elephant seals. This results support the hypothesis that SES prey tend to not aggregate in large discrete schools. Indeed, in such a situation the predator is expected to adopt an overall sinuous and spherical trajectory. A spherical first passage time (SFPT, [71]) analysis performed on our dataset revealed that “Volume-restricted search” (VRS) could be detected in the bottom of half of the SES dives in which case they accounted for 36.9% of the bottom phase duration but 67.6% of PEE [72]. The typical scale of VRS, 48.2±25.7 m, was similar for all individuals and the average prey encounter rate was 1.2±*S* *D*=0.3 PEE min^-1^ inside VRS and 0.3±0.2 PEE min^-1^ outside VRS [72]). These results indicate that the prey density may vary at finer scales than the one typically investigated in this study (entire bottom phases), but not by a very large amount. Moreover, the VRS were (mechanically) related to a decrease of the SES swimming speed and greater path sinuosity but the SES trajectories remained dominated by a single direction. These patterns of the 3D path of SES at the bottom of their dives suggest that the deep scattering layer consists of dispersed solitary prey or small group of individuals.

### PEE density along depth

The dominant direction in the trajectories of SES at dives’ bottom was primarily horizontal (Fig. 4). This indicates that SES target specific layers of the water column during the bottom phase of their dive [73, 74]. Shallower layers require less time and energy to be reached by SES and, therefore, are more accessible and more profitable. Performing dives’ bottom phase at greater depth was found to be associated with smaller prey encounter density (Table 5). While the SES and their prey perform diel vertical migrations [6, 8–10], this process cannot explain our result because all the 3D dives in our dataset occurred during daytime. A previous study, based on likelihood of detecting bioluminescence [75] highlighted a similar pattern. The impact of the changes in the size or species composition of the mesopelagic community in relation with this decreasing prey encounter density could not be examined with the tools available to us but they could have substantial importance. Assuming that the energy content of a prey item does not vary with depth, the deep dives would imply smaller energy income (because of reduced encounter rates) but larger expenditure (because of the longer transit between surface and bottom). Thus, these dives would be be doubly detrimental to the SES energy balance. However, the probability that no PEE occur decreased with the SES diving depth (Table 5), suggesting that a better resource predictability in deep waters might compensate to some extent for this energy shortfall. SES were found to dive deeper north of the Sub-Antarctic Front but yet to maintain their mass gain which suggesting that they could target larger and/or richer prey [25]. Similarly, SES could expand their diet to other prey types and/or sizes when foraging at depth, allowing steadier prey catch rate and ensuring a baseline level of energy intake.

We distinguished between two sources of vertical exploration in the explanatory variables: depth range covered by SES (1) by moving along the first main component and (2) by moving orthogonally to this component. An increase in any of these was associated with a decrease of the prey density proxy (Table 5). The amount of horizontal exploration orthogonally the main direction of the bottom trajectory was also negatively related to the prey density encountered but to a lesser degree than observed in the vertical dimension (Table 5). Not only the SES target specific layers, but the prey density in this layer is primarily determined according to the vertical dimension. This result suggest that local prey density could by driven by vertical constraints that delineate the vertical extent of the deep scattering layer. The nature of these constraints could be biotic (e.g. predation risk, aggregation into reproductive swarms) or abiotic (e.g. habitat preferences regarding temperature, light intensity or oxygen concentration). For instance, harbour seals (*Phoca vitulina*) have been reported to adjust their diving depth to in relation to the mixed layer depth [74]. In addition to the effect of oceanographic parameters on the prey abundance, such conditions could impact locally on the foraging success of SES by modulating the prey density [73, 76].

A trade-off between feeding resources richness (primary production taking place in the well-lit sub-surface water) and predation risk (expected to be greater in luminous environment) is responsible for the diel vertical migration pattern phenomenon [77]. The light level intensity, decreasing with depth, delineate the upper boundary of many pelagic species distribution and, consequently, relates to the diving depth of SES [10]. Because diving predators are constrained to return to the surface in order to breathe, they do not benefit from pursuing deeper when an exploitable prey patch is encountered. With bio-logging, data sampling relies on the decisions of free-ranging animals [78]. The so collected presence-only data makes it difficult to assess the deeper limit of the prey patch on which the SES forage. Therefore, the relationship between the PEE density and the vertical extent of the SES path underwater conveys more qualitative than quantitative information about the link between thickness of the deep scattering layer and its corresponding density.

### Foraging behaviour

The prey density proxy was negatively related to an increase of the overall travel distance in the bottom phase (total dispersion, Table 5). In high density patches, the SES would travel shorter overall distances which could be explained by a greater locomotion cost, more active swimming behaviours related to hunting strategy or prey pursuing could force them to end the bottom phase early. Dominance of the first main component (MC1 dispersion, Table 5) which corresponds predominantly to horizontal movements (Fig. 4) was associated with smaller PEE density (18 m model, Table 5). This results indicates that SES trajectories in denser prey environment tend to be slightly less linear. However, this effect was not consistently observed across all models.

On the whole, horizontal exploration mainly takes place moving forward according to the first main component. Based on their observation of the behaviour of *Thunnus maccoyii*, [79] suggested that feeding during periods of straight movement could be more common than expected from the optimal foraging theory. At the scale of a complete foraging trip at sea, SES feeds to a large extent during the transit part of their trip but this pattern seems to be observable within the dives’ bottom phase where foraging is expected to be the primary objective of the diving predator. Such an extensive-search behaviour is expected when prey are well dispersed in the environment [80] which seems consistent with the suspected non-schooling behaviour of SES prey that we have previously discussed.

Sensory perception of the surrounding environment has direct consequences on the predator-prey interactions as it mediates animal’s ability to locate prey and/or escape predation. The notion of prey dispersion is thus relative to the sensory detection range of the predator. While the scale of this perception in natural conditions is largely unknown for elephant seals, more information about the senses involved are available from functional anatomy and pool experimentations on northern elephant seals. Northern and southern elephant seals forage at great depth and thus, under very dark conditions [81]. Peak sensitivity of their vision, occurring at around *λ*=485 nm, is adapted to a spectrum of low light intensity [82] and bioluminescence [75] such as that emitted by some of their myctophid prey. Additionally, elephant seals possess enhanced visual sensitivity and rapid adaptation to darkness [83]. Like many other pinnipeds, elephant seals have highly sensitive whiskers [81], that repeatedly protract before prey captures [30]. However, while it can be assumed that these senses (vision and tactile) are used to locate prey, the extent of their spatial coverage is unclear. Very little information about the auditory capacity of elephant seals is available. It is known that pinnipeds do not echolocate [84] but, as myctophids can emit sound, elephant seals could use passive audition instead. Finally, a recent study of the 3D underwater path of northern elephant seals [45] highlighted volume-restricted search spatial-scales of 8–10 m and 17–19 m, possibly related to prey distribution and/or perception range of the predator. On that basis, we considered a wide range of prey detection distance, 1.5, 9 and 18 m (according to [45]), to define the boundaries of water volume within which we hypothesize that SES could detect the presence of prey items. If the spread of prey items largely exceeds the range of the predator a straight path is an efficient sampling strategy to scan large volumes of water. In view of the strong linear trend exhibited by the SES in their 3D path, our results bring best supports to a short detection distance scenario.

Besides the hypothesis that behaviour is driven by the prey distribution, active feeding in travel could be due to migratory constraints. Indeed, such a situation could result from the evolution of migratory opportunistic predators that need to meet its energetic requirements while moving rapidly [79]. Given the very wide range of oceanographic conditions the elephant seal explore [60], the opportunistic behaviour may a be relevant point. However, it is unclear if the migratory constraint applies to SES. Indeed, the primary goal of their trip at sea is believed to be foraging but they do so to a greater or lesser extent all along their trip wandering about 43 km day^-1^ in intensive foraging and 75 km day^-1^ otherwise [85].

Our estimates of the prey density changed very quickly according to the chosen detection radius ranging in four orders of magnitude from a few *μ*PEE m^−3^ to hundreds *μ*PEE m^−3^. Sampling micronekton with a large mid-water trawl, [86] found an average micronekton biomass of 2.5×10^−03^ g m^−3^ during the day (250 *μ*PEE m^−3^ considering an average fish weight of 10 g). Furthermore, [87] estimations of micronekton density ranged from 0 to 6000 *μ*PEE m^−3^. Among the detection radius we tested, the 1.5 m radius yields the closest results (715±*S* *D*=760 *μ*PEE m^−3^, Table 4). Our estimation of the prey density rely on the idea that SES do attempt to catch a prey when they detect one. The prey avoidance as well as the probability of multiple simultaneous prey encounters could not be taken into account. These special cases however seem less likely in a short detection distance scenario such as a 1.5 m radius. Despite the correspondence between the amount of PEE detected from head-mounted and back-mounted acceleration data [62, Additional file 2], the latter have a tendency to miss some events. As a result we expect our estimations of the prey encounter density to be underestimated. We suggest that the hunting tactics of SES may be opportunistic in the sense that prey item would be detected at short distances and suddenly be captured without substantial chase ([72]).

### Limitations of the study

Each method used to assess the micronekton resources of the pelagic ecosystem have their own weaknesses. Trawl sampling allows identification of size and species but is costly [88], requires good weather operating conditions and net avoidance of the different species are still unknown but highly expected [89]. Bio-logging implies a bias sampling due to different range of habitat available to the predators and prey (a typical example for diving predators is the depth range) and difficulties to distinguish between what is related to animal behaviour and to the environment. For instance, the predator decision to attack a prey can involve many parameters such as the type of prey, its size, its energetic content, its handling time, and abundance. As such, implicit hypothesis are often made to simplify animal behaviours interpretation: animals are assumed to be always efficient to catch their prey; the potential effect of nearby predators on the behaviour is neglected etc... Eventually, bio-logging studies are also limited by the number of individuals that could be equipped [90] and generally lack the information on prey species. Acoustic surveys depend on presence/absence of a swim bladder as well as on its composition (gas or lipid) which, for some species, is known to change according to the stage of development. Distinguishing between species and estimating biomass is thus difficult with communities of mixed species and/or mixed ages. Spatial resolution of the data also decrease with depth as lower signal frequencies are required. In this context, pairing these approaches – for instance by deploying sonar tags or synchronizing in space and time the trawling survey with predators feeding areas – could greatly assist the scientist to better understand the micronekton ecology.

Because of the small number of individuals for which we could reconstruct 3D path (six), it is still unclear if the diving behaviour we observed extends at the population level. Concerning the negative effect of (i) the mean depth and (ii) the vertical spread of the bottom phases on the estimated prey encounter density, it is to be noted that similar relationships have been highlighted on nine other individuals [62]. The strong dominance of a single direction in the elephant seal path was consistently observed for all our individuals, but this trend has not been previously reported for southern elephant seal. Nonetheless, [41] highlighted a similar pattern on a single northern elephant seal (20 dives). Stronger evidence on the prevalence of these behaviours may accumulate as 3D path analysis will develop in the future.

Due to the limited Acousondes^TM^ battery life, we could only sample the first part of the SES foraging trips, where they tend to adopt a faster horizontal transit rate Due to this sampling bias, our results could overstate the dominance of linear horizontal paths at the bottom of dives. We found that higher prey density are associated with shorter bottom phases (Total dispersion, Table 5). Furthermore, foraging dives of SES are characterized by steep pitch angle in descent and ascent phases, minimizing horizontal displacement [91]. These factors could explain the correlation between transit rate slowing and SES prey density better than changes of the bottom path sinuosity. Further studies could analyse underwater 3D trajectories in other parts of SES trip to overcome the sampling bias of our study and adjudicate this issue. Area-restricted search are supposedly periods where SES meet high prey densities and are likely to exhibit larger horizontal sinuosity in their bottom phase 3D path. As such, the ARS appear like interesting periods to address this particular issue.

The 3D reconstruction of the SES path underwater by dead-reckoning assumes that the direction of travel of the animal is always parallel to the body orientation. More sophisticated methods such as the one developed by [92] which is free of this assumption, attest that this approximation can have an impact on the reconstructed tracks. However, such methods require large computation times and are not suitable for datasets of several hundreds of dives. Dead-reckoning is also subject to cumulative errors. Therefore, uncertainties about the shape of the SES trajectories at the bottom of their dives increase with the dive depth and the duration of bottom phase. Conversely to other methods ([54, 92]), our method did not estimate position uncertainties. So, we could not account for its effects in the analysis of the 3D trajectories. The last discussion point about the 3D reconstruction method implemented in this study relates to the assessment of SES swimming speed. The water-flow noise to water-flow speed relationship in the descent phase, extrapolated to entire dives to estimate SES swimming speed, presuppose that the flow behaves similarly throughout the dives. This is yet to be verified.

### Perspectives

The straightness of the SES underwater path has been reported by [41] who highlight its consistency from one dive to another. It is not clear how SES orientate themselves and how migratory objectives contribute to this pattern. Examining this pattern in relation to currents [93] would be interesting in order to study the navigation skill of SES.

We could only focus on the quantitative aspect of prey field because information about the nature of PEE was not available. Thus, it is unclear if the results mainly concern one type of prey such as a specific myctophid species more abundant in the study area – the eastern edge of the Kerguelen shelf – or partially apply to the different prey types targeted by SES. We could not test for the role of the quality of prey items (size and species) which could imply distinct types of predator-prey spatial interaction due to different detectability, aggregative behaviour or predator-escaping abilities (responsiveness, speed, maneuverability). To address these issues a camera is needed in order to identify the species and size of the prey items [28, 30, 94]. However, the high power consumption of these devices and the very dark environment the SES forage in are still technical constraints to their usage. Miniaturized sonar [95] could bring the power of high-frequency acoustic signals to identify prey type at new depths. In quantitative terms it is also promising tool: by extending the perception range of bio-logging outside of the very intimate sphere surrounding the animals it could allow to examine thoroughly the fine-scale prey distribution, the range and mechanisms of the prey detection and hunting tactics of SES.

## Conclusion

Analysis of main components of 3D SES paths in their dives’ bottom phase allowed us to describe the main trends in SES movements in these key periods of foraging. Such an approach, examining the overall use of space, may be complementary to ARS/VRS analysis that focus on the most sinuous part of the the animal paths, and conveys new information on predator-prey interactions.

The 3D space use of SES at the bottom of their dives suggests that prey do not tend to form large discrete schools but rather adopt a scattered distribution structured in layers. The prey encounter density in these layers decreased with depth but then, SES tended to exhibit prey encounter events on a more regular basis. However, it is not clear how to interpret this tendency given that qualitative information of the prey (size and species) is missing. We suggest that the prey density decrease with depth but that their distribution tends to standardize yielding higher predictability. The extent of the vertical exploration performed by the SES during their bottom phases related negatively to prey encounter density, seemingly indicating that the thickness of the layers targeted by SES mechanically impacts micronekton density. These results underline the primary importance of the vertical dimension into the spatial organization of the micronekton.

The 3D trajectories in our dataset were essentially linear paths. While the vertical deviations from this path were of the the same order of magnitude of the horizontal ones, they better related to the prey encounter density. Under such circumstances, the widely used time-depth recorders can be considered as an effective simplification of the SES movements at scales of few-hundreds meters. Adaptive mechanisms underlying this behaviour, such as a trade-off between the travel speed and energetic requirements or an unbalanced ratio between SES sensory perception range and prey distribution and avoidance, remain unclear. These observations could be related to a number of combined factors: prey field organized in layers, short prey detection distance, external constraint such as the purpose for SES to move away rapidly from their breeding site.

We believe that this study highlights the importance of knowledge about the three-dimensional predator-prey interactions and gives support to the usage of bio-logging to unravel and monitor fine-scale micronekton distribution, particularly in remote areas such as the deep pelagic ecosystems of the Southern ocean.

## Additional files

Additional file 1 Assessment of prospected water volume. The Monte Carlo integration provided an easy-to-implement method in order to estimate the volume of water surrounding the trajectory of southern elephant seals. However this numeric method is sensitive to the sampling effort as well as to the chosen detection radius. Here, we present the code and results of a simple experiment to quantify the uncertainty of water volume estimates with the settings used in the paper. (PDF 403 kb)

Additional file 2 Partial regression lines for 1.5 m and 9 m radii models Additional figures to present the results of models with 1.5 m and 9 m radii. (PDF 673 kb)

## Abbreviations

1D,2D,3D: Uni-dimensional, Bi-dimensional, Tri-deminesional
AIC: Akaïke information criterion
ARS: Area-restricted search
CV: Coefficient of variation
CI: Confidence interval
GPS: Global positioning system
MC1: First main component, also called first main component
n: number of
PEE: Prey encounter event
SD: Standard deviation
SE: Standard error
SES: southern elephant seals
VRS: Volume-restricted search
