# Supplementary material for: Three-dimensional space use during the bottom phase of southern elephant seal dives
Source: Mov Ecol. 2017 Aug 31;5:18. doi: 10.1186/s40462-017-0108-y (PMC5577837; doi:10.1186/s40462-017-0108-y)
Supplement: Supplementary file 1 — Assessment of prospected water volume. The Monte Carlo integration provided an easy-to-implement method in order to estimate the volume of water surrounding the trajectory of southern elephant seals. However this numeric method is sensitive to the sampling effort as well as to the chosen detection radius. Here, we present the code and results of a simple experiment to quantify the uncertainty of water volume estimates with the settings used in the paper. (PDF 403 kb) [file 40462_2017_108_MOESM1_ESM.pdf]

“Water volume prospected by elephant seals”

Monte Carlo Integration: motivations and principle

We called “volume of water prospected by SES” the volume of water where the SES would have been able to locate prey items. The longer is the SES path, the larger is this volume. However, this volume also depends (i) on the range of the SES perception, and (ii) on the tortuosity of the trajectories. Similarly to the two-dimentional case at surface where tortuous tracks are associated with “Areas of Restricted Search”, increasing 3D paths tortuousity implies prospection of smaller water volumes.

Because the SES path at the bottom of their dives can have complex shapes it is not possible to apply a general formula. The convex hull volume did not yield satifying results. Indeed, it appeared that the convex full volume greatly varies according to the location of a few points at the extremities of the trajectories, and it does not take into account the limitated abilities of SES to locate prey at great distances. Hence, we used a numerical method called Monte Carlo Integration (MCI) to assess the volume of water prospected by SES. A simple illustration of the principle of this method in 2D is presented on the figure 1 taken from wikipedia.

To implement MCI assessment of the volume prospected by SES at the bottom of dives, we made the assumption that SES could locate prey all around them, up to a given distance which is hereafter called “detection radius”. The calculation of the volume is then a simple application of the example presented on the figure 1 with a third dimension: the square becomes a cuboid that encompass the trajectory of SES and whose volume is easy to calculate, the circle becomes a sphere whose radius is equal to the detection distance. The prospected water volume is calculated by taking the volume of the cuboid multiplied by the proportion of random points located within a given distance (dtection radius) from *any* specific SES location.

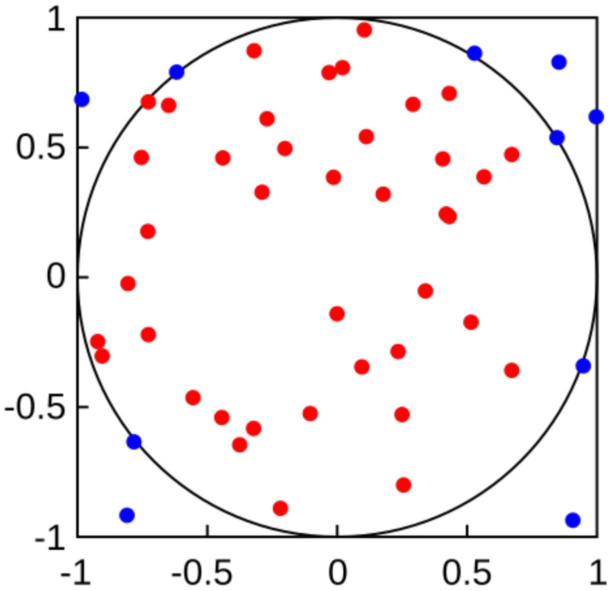

Figure 1: Illustration of Monte Carlo integration to assess π. Figure taken from the Wikipedia article entitled "Monte Carlo integration". Original caption: "In this example, the domain D is the inner circle and the domain E is the square. Because the square’s area (4) can be easily calculated, the area of the circle ($\pi * 1^2$) can be estimated by the ratio (0.8) of the points inside the circle (40) to the total number of points (50), yielding an approximation for the circle’s area of $4 * 0.8 = 3.2 \sim \pi * 1^2$"

R code implementation

```r
#' Assessement of prospected volume underwater using Monte Carlo integration
#'
#' @param x A data frame with the 3D coordinates of the animal locations (in meters).
#' @param n_sample Number of random points to use, higher values yield more accurate estimates.
#' @param radius Detection radius (in meters).
#' @return Estimate of the prospected volume in m^3
MCi_volume <- function(x, n_sample, radius) {
  stopifnot(require("fields")) # rdist function
  stopifnot(require("rbl"))    # "first" & "last" functions
  # Get limits of the cuboid enclosing bottom track +- "radius"
  cuboid_limits <- Map("+", lapply(x, range), list(radius * c(-1,1)))
  cuboid_volume <- prod(sapply(cuboid_limits, diff))
  # Generate uniformely distributed random points in the cuboid
  random_pts <- as.data.frame(
    Map(runif,
        n = n_sample,
        min = sapply(cuboid_limits, first),
        max = sapply(cuboid_limits, last))
  )
  # Proportion of random points located within "radius" m from any track location
  dist_matrix <- rdist(x, random_pts)
  pts_withinVol_proportion <- mean(apply(dist_matrix <= radius, 2, any))
  cuboid_volume * pts_withinVol_proportion
}
```

Reliability of the method according to detection radius and sampling effort

Experiment setup

Here, we perform an experiment to visualize how volume estimates vary (i) from a dive to another, (ii) according to the sampling effort, (iii) according to the detection radius.

- We select the bottom phases of a random sample of 48 dives from our dataset.
- For each of these bottom phases and each detection radius (1.5, 9 and 18 meters), we assess the volume of water prospected along the 3D path of SES 25 times using MCI and various sampling effort. This allows us to visualize how the the means and variances of the prospected water volume estimates behave according to the sampling effort, for a given radius and a given bottom phase.

Results of the experiment

The figure 2 presents the raw results (volume estimates) for the 9 m radius, using boxplots. As expected, the sampling effort used during the MCI has a great impact on the variance of the estimates but a lower impact on the mean/median estimation. The MCI method is unbiased, but its accuracy is very sensitive to the sampling effort.

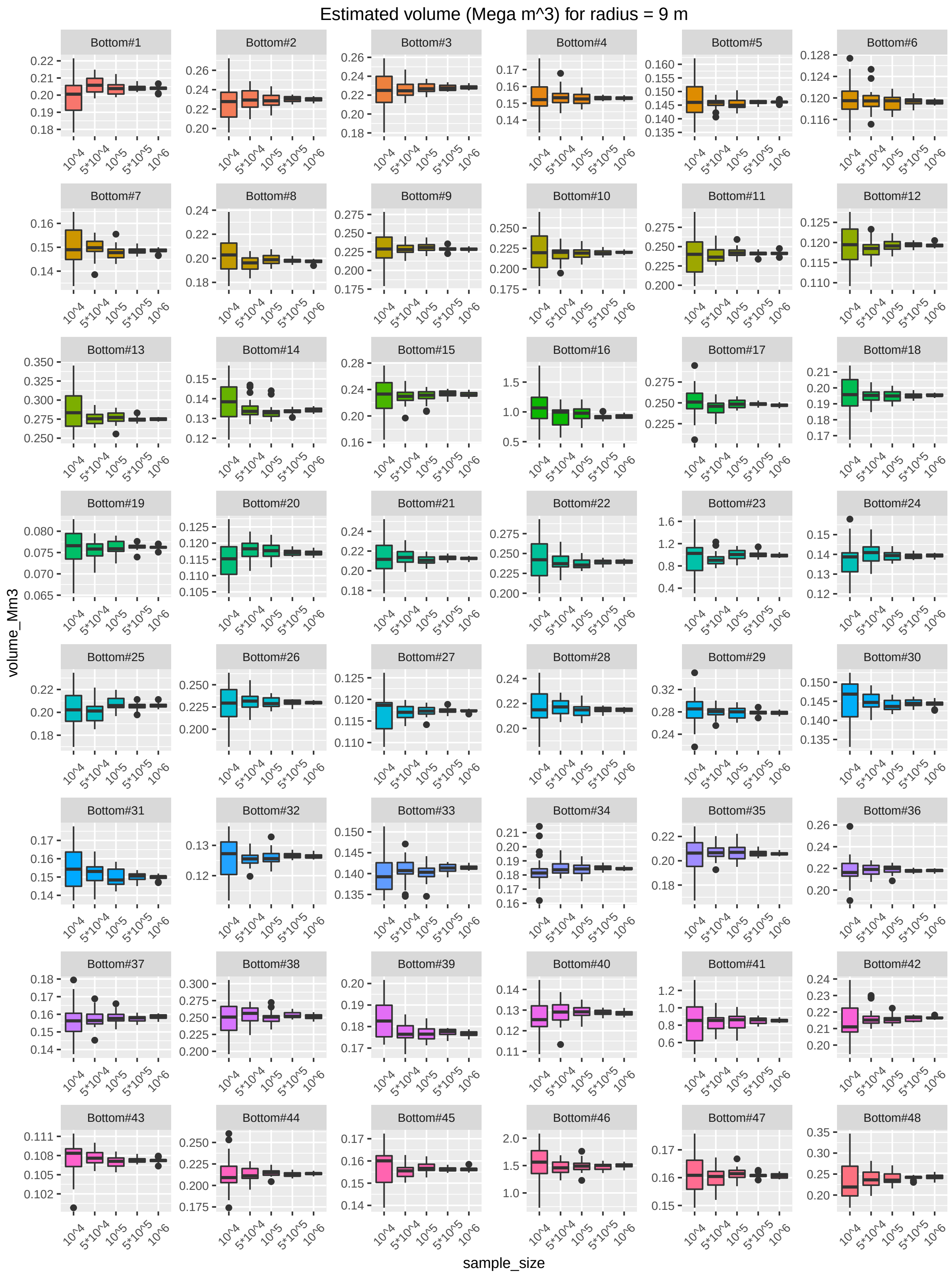

Figure 2: Boxplots of the estimated prospected volumes according to the number of random points used in MCI (radius = 9 m). Notice that X axis is in log-scale and that Y axis scale varies according to dive ID.

The results of the entire experiment are summarized on figures 3 and 4 using the variation coefficient (sample standard deviation / sample mean), which can be considered as the measurement error of the MCI method. There is roughly a linear relationship in log-log scale between the sampling effort and the method accuracy. The intercept of the relationship depends on the detection radius and on the bottom phase whose volume is being assessed. The detection radius has a large impact on the variation coefficient, with larger radius yielding better accuracy. This highlights that higher sampling effort is necessary for the smallest radius we used in the study (1.5 meter). The slopes of the relationships are similar between the various detection radius and bottom phases. These graphics allowed us to adjudciate that a sampling effort of 500,000 points was sufficient for the 9 and 18 meter radius. This sampling effort was doubled for the 1.5 meter radius. The next sections provide additional details on this subject.

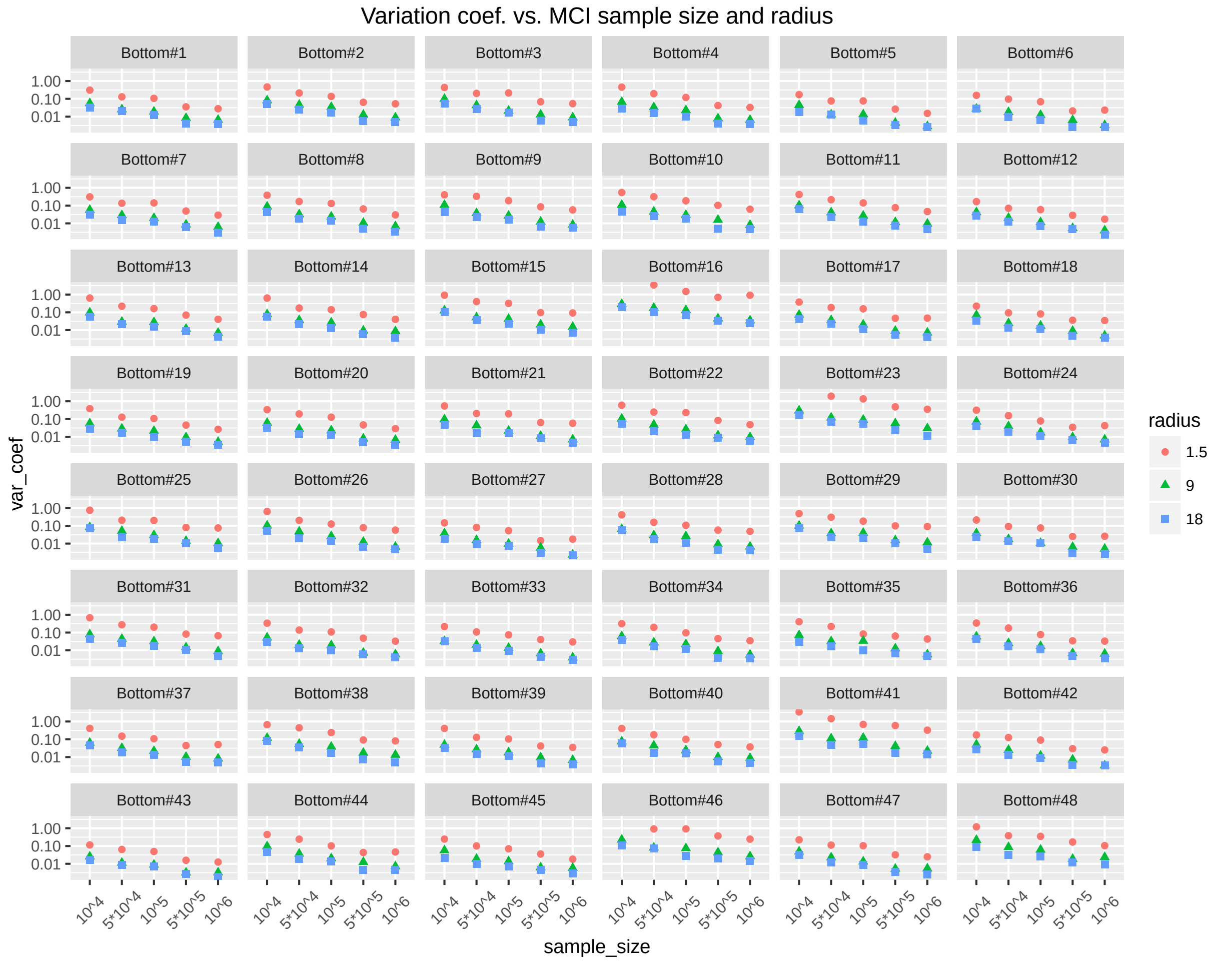

Figure 3: Variation coefficient of the estimated prospected volumes according to the number of random points used in MCI, and according to the detection radius. X and Y axis in log-scales (base 2 and base 10).

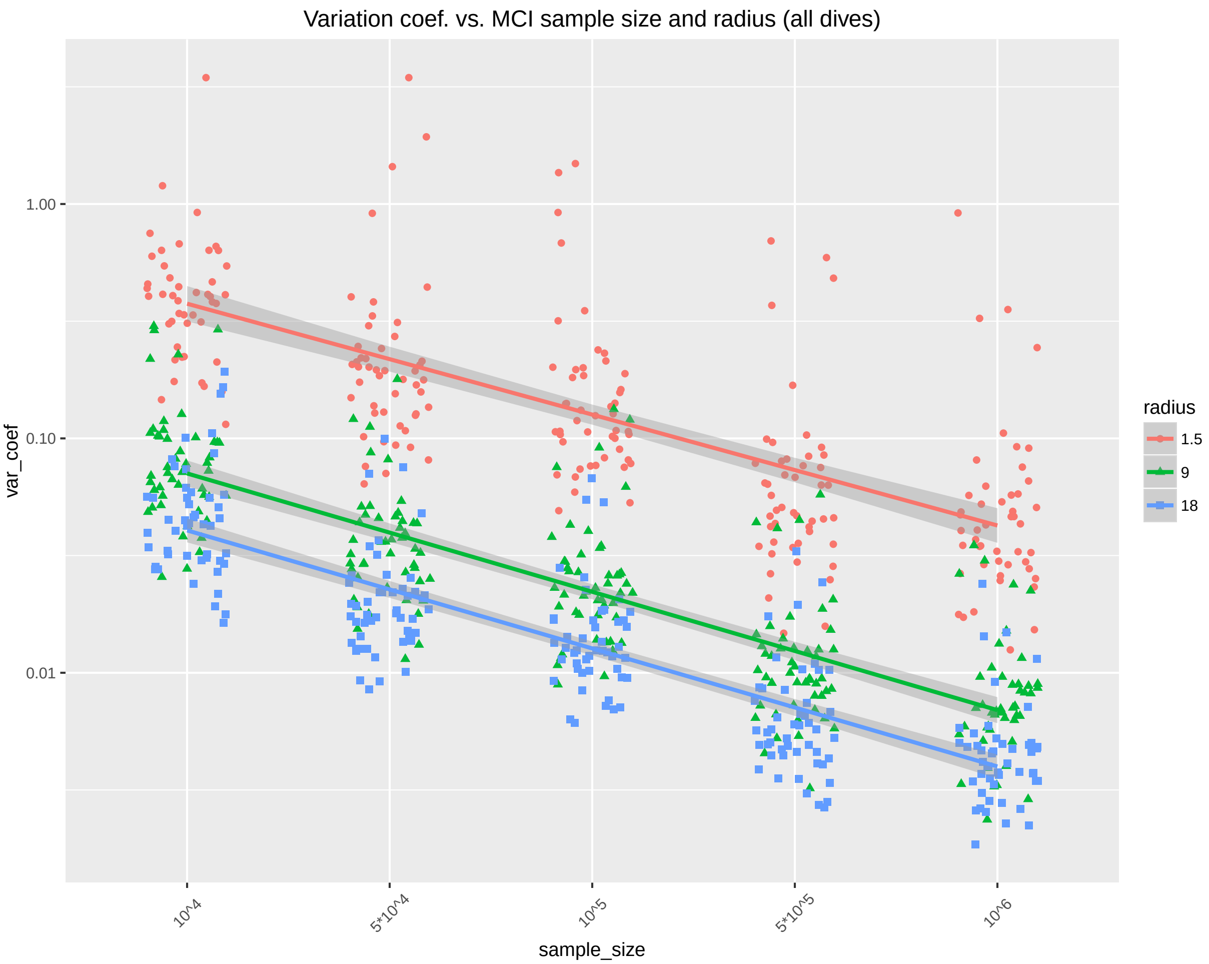

Figure 4: Variation coefficient of the estimated prospected volumes according to the number of random points used in MCI, and according to the detection radius. X and Y axis in log-scales (base 2 and base 10). Some horizontal jitter have been added to avoid points to overlap and provide better readalibity

Coefficient of variation for the settings used in main paper analyses

This section provides summary statistics (mean and 95 % quantile), as well as raw numeric values, of the variation coefficient obtained for MCI settings used in the study (tables 1, 2 and 3). These results reveal that prospected volume estimates are accurate for n = 500000 random points in the MCI if the detection radius is at least 9 meters (tables 1 & 2). For the radius = 1.5 m, the sample size has to be doubled to reach a decent accuracy (table 3).

Table 1: Values for radius = 18 m, n = 5*10^5 random points, NA indicates that all replicates estimated volume to 0. Mean variation coefficient = 0.0073, Quantile 95% = 0.0188

bottom_id	var_coef	bottom_id	var_coef	bottom_id	var_coef
Bottom#1	0.004101	Bottom#17	0.005722	Bottom#33	0.00444
Bottom#2	0.005668	Bottom#18	0.004919	Bottom#34	0.003878
Bottom#3	0.006016	Bottom#19	0.005257	Bottom#35	0.006803
Bottom#4	0.004061	Bottom#20	0.004919	Bottom#36	0.004885
Bottom#5	0.003396	Bottom#21	0.008449	Bottom#37	0.005278
Bottom#6	0.002665	Bottom#22	0.008656	Bottom#38	0.007579
Bottom#7	0.006112	Bottom#23	0.02433	Bottom#39	0.004309
Bottom#8	0.00504	Bottom#24	0.006446	Bottom#40	0.005574
Bottom#9	0.006666	Bottom#25	0.01031	Bottom#41	0.01745
Bottom#10	0.004944	Bottom#26	0.006556	Bottom#42	0.003551
Bottom#11	0.007439	Bottom#27	0.003053	Bottom#43	0.002736
Bottom#12	0.004713	Bottom#28	0.004591	Bottom#44	0.004435
Bottom#13	0.0086	Bottom#29	0.01029	Bottom#45	0.004606
Bottom#14	0.005738	Bottom#30	0.002811	Bottom#46	0.01946
Bottom#15	0.01035	Bottom#31	0.01093	Bottom#47	0.003518
Bottom#16	0.03303	Bottom#32	0.005955	Bottom#48	0.01164

Table 2: Values for radius = 9 m, n = 5*10^5 random points, NA indicates that all replicates estimated volume to 0. Mean variation coefficient = 0.0132, Quantile 95% = 0.043

bottom_id	var_coef	bottom_id	var_coef	bottom_id	var_coef
Bottom#1	0.00835	Bottom#17	0.009097	Bottom#33	0.006644
Bottom#2	0.01269	Bottom#18	0.009049	Bottom#34	0.008996
Bottom#3	0.01298	Bottom#19	0.009441	Bottom#35	0.01274
Bottom#4	0.007965	Bottom#20	0.007978	Bottom#36	0.006943
Bottom#5	0.004534	Bottom#21	0.01108	Bottom#37	0.01025
Bottom#6	0.006385	Bottom#22	0.01205	Bottom#38	0.01734
Bottom#7	0.008539	Bottom#23	0.05756	Bottom#39	0.009557
Bottom#8	0.01062	Bottom#24	0.009453	Bottom#40	0.009992
Bottom#9	0.01237	Bottom#25	0.014	Bottom#41	0.04134
Bottom#10	0.01581	Bottom#26	0.01257	Bottom#42	0.007243
Bottom#11	0.01174	Bottom#27	0.005787	Bottom#43	0.003215
Bottom#12	0.005374	Bottom#28	0.00908	Bottom#44	0.01256
Bottom#13	0.0118	Bottom#29	0.01524	Bottom#45	0.006279
Bottom#14	0.009319	Bottom#30	0.006417	Bottom#46	0.04384
Bottom#15	0.02054	Bottom#31	0.01458	Bottom#47	0.005257
Bottom#16	0.04485	Bottom#32	0.007271	Bottom#48	0.01874

Table 3: Values for radius = 1.5 m, n = 10^6 random points, NA indicates that all replicates estimated volume to 0. Mean variation coefficient = 0.0779, Quantile 95% = 0.2967

bottom_id	var_coef	bottom_id	var_coef	bottom_id	var_coef
Bottom#1	0.02778	Bottom#17	0.0472	Bottom#33	0.02973
Bottom#2	0.05241	Bottom#18	0.03461	Bottom#34	0.0348
Bottom#3	0.0536	Bottom#19	0.0265	Bottom#35	0.04319
Bottom#4	0.03284	Bottom#20	0.0289	Bottom#36	0.03298
Bottom#5	0.01527	Bottom#21	0.05794	Bottom#37	0.05072
Bottom#6	0.02323	Bottom#22	0.04855	Bottom#38	0.08086
Bottom#7	0.02895	Bottom#23	0.3546	Bottom#39	0.03495
Bottom#8	0.02995	Bottom#24	0.04275	Bottom#40	0.03699
Bottom#9	0.05712	Bottom#25	0.07542	Bottom#41	0.3251
Bottom#10	0.06254	Bottom#26	0.05729	Bottom#42	0.02522
Bottom#11	0.0464	Bottom#27	0.01773	Bottom#43	0.01253
Bottom#12	0.01724	Bottom#28	0.04881	Bottom#44	0.04635
Bottom#13	0.04065	Bottom#29	0.09085	Bottom#45	0.01819
Bottom#14	0.04043	Bottom#30	0.02593	Bottom#46	0.244
Bottom#15	0.09213	Bottom#31	0.06581	Bottom#47	0.02475
Bottom#16	0.916	Bottom#32	0.03257	Bottom#48	0.1053

Influence of the dive bottom phase ID with mixed models

We implemented a selection of mixed models in order to focus on the impact of the dive ID on the variation coefficient (CV), given a detection radius and sample size in MCI.

Conclusions:

- We found that dive ID only impact the intercept of the CV-Sample size relationship, that is the base level of the CV, but not the speed at which the CV decreases with inceasing sample size. When sample size is multiplied by 10 ($+1 = log_{10}(10)$), CV is approximatively divided by 3 ($-0.5 \sim log_{10}(1/3)$, see fixed effects summary and figure 4 for their graphical representation). Extrapolating this relationship, it appears that it is not technically achievable to us (due to computation time and RAM) to increase the sample size for the smallest radius so that the corresponding CV reaches the level obtained for other radii.
- Additionaly, the random effect of the dive ID is much larger for the small radius (1.5 meter, std. dev. = 0.34), for which the intercepts are about three times more variable (see random effects summary and tables 1 2 and 3). As a consequence the distance between the average variation coefficient and the 95% quantile is larger for the 1.5 meter detection radius (tables 1, 2 and 3).

Details:

From figures 3 and 4, the list of model to test is quite small. Here are provided the details about the model selection and the model estimates for the best candidate.

```
# Selecting random part of the mixed model with full fixed effects
# No random effect
full.random0 <- lm(log10_var_coef ~ 1 + radius * log10_sampling,
                       data = vol_stats)
# Random intercept only
full.random1 <- lmer(log10_var_coef ~ (1 | bottom_id)
                         + radius * log10_sampling,
```

```r
                         data = vol_stats, REML = FALSE)
# Random log10_sampling slope and intercept
full.random2 <- lmer(log10_var_coef ~ (1 + log10_sampling | bottom_id)
                         + radius * log10_sampling,
                         data = vol_stats, REML = FALSE)
# Random radius "slope" and intercept
full.random3 <- lmer(log10_var_coef ~ (1 + radius | bottom_id)
                         + radius * log10_sampling,
                         data = vol_stats, REML = FALSE)
# Random radius & log10_sampling slopes and intercept
# This model fails to converge: "degenerate Hessian with 1 negative eigenvalues" warning
full.random4 <- lmer(log10_var_coef ~ (1 + radius + log10_sampling | bottom_id)
                         + radius * log10_sampling,
                         data = vol_stats, REML = FALSE)
# Compare AIC
AIC(full.random0, full.random1, full.random2, full.random3, full.random4)
```

```
##              df        AIC
## full.random0  7   176.5287
## full.random1  8 -1208.3033
## full.random2 10 -1207.0704
## full.random3 13 -1503.2449
## full.random4 17 -1497.3354
```

```r
# Select fixed effects using random effect structure #3
nointeraction.random3 <- lmer(log10_var_coef ~ (1 + radius | bottom_id)
                                  + radius + log10_sampling,
                                  data = vol_stats, REML = FALSE)
# Compare AIC
AIC(full.random3, nointeraction.random3)
```

```
##                       df       AIC
## full.random3          13 -1503.245
## nointeraction.random3 11 -1506.798
```

```r
# Print final model estimates
mod.final <- update(nointeraction.random3, REML = TRUE)
summary(mod.final)
```

```
## Linear mixed model fit by REML ['lmerMod']
## Formula:
## log10_var_coef ~ (1 + radius | bottom_id) + radius + log10_sampling
##    Data: vol_stats
##
## REML criterion at convergence: -1498.4
##
## Scaled residuals:
##      Min       1Q   Median       3Q      Max
## -2.70544 -0.62681  0.03389  0.64690  2.98103
##
## Random effects:
##  Groups    Name        Variance Std.Dev. Corr
```

```
##  bottom_id (Intercept) 0.116178 0.34085
##            radius9     0.011504 0.10726  -0.95
##            radius18    0.015822 0.12579  -0.99  0.99
##  Residual              0.004646 0.06817
## Number of obs: 717, groups:  bottom_id, 48
##
## Fixed effects:
##                Estimate Std. Error t value
## (Intercept)     1.667126   0.052655   31.66
## radius9        -0.768467   0.016694  -46.03
## radius18       -1.010172   0.019200  -52.61
## log10_sampling -0.502632   0.003579 -140.44
##
## Correlation of Fixed Effects:
##             (Intr) radis9 rads18
## radius9     -0.848
## radius18    -0.894  0.926
## lg10_smplng -0.346  0.003  0.003
```

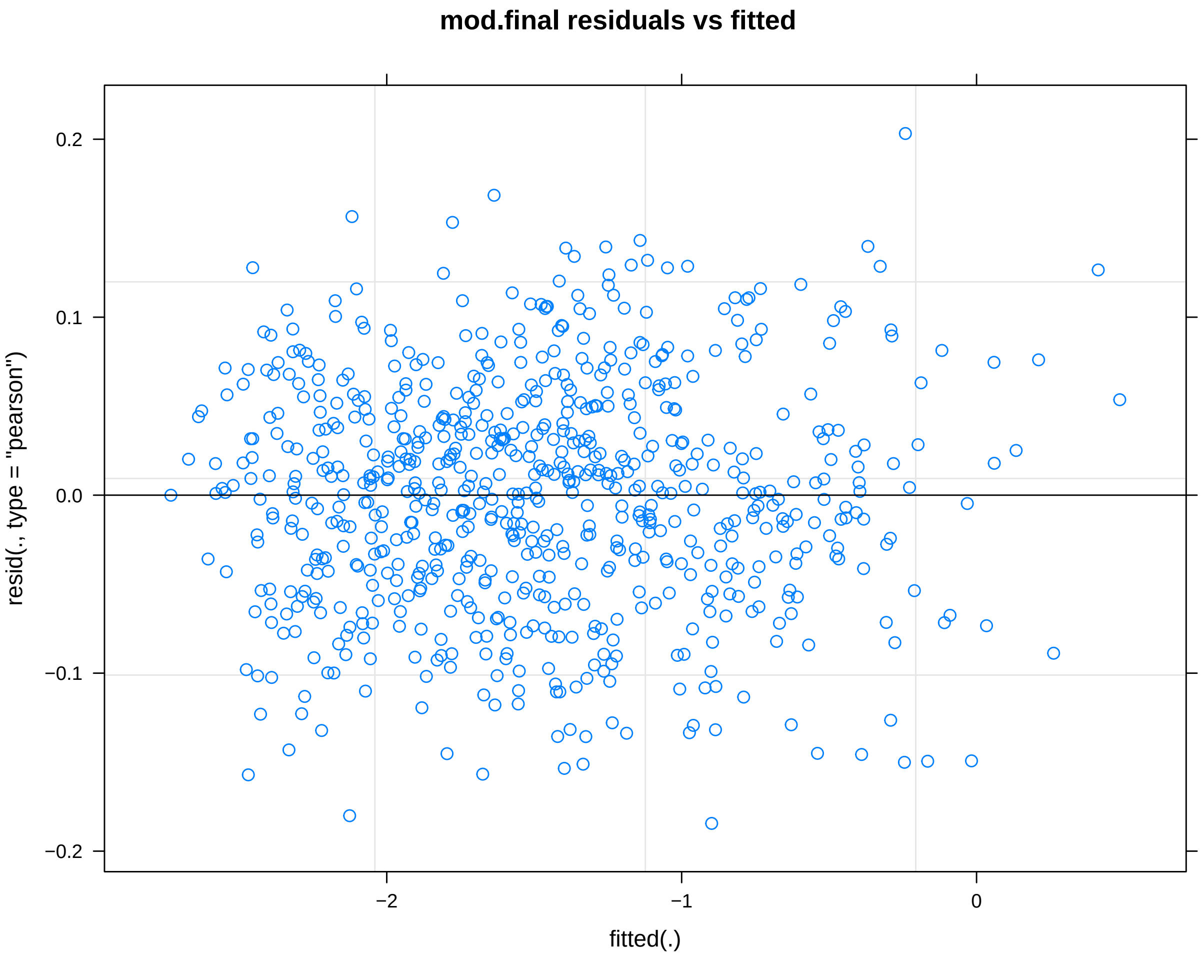

Figure 5: Model residuals
