# Supplementary material for: Three-dimensional space use during the bottom phase of southern elephant seal dives
Source: Mov Ecol. 2017 Aug 31;5:18. doi: 10.1186/s40462-017-0108-y (PMC5577837; doi:10.1186/s40462-017-0108-y)
Supplement: Supplementary file 2 — Partial regression lines for 1.5 m and 9 m radii models Additional figures to present the results of models with 1.5 m and 9 m radii. (PDF 673 kb) [file 40462_2017_108_MOESM2_ESM.pdf]

Partial regression lines for 1.5 m and 9 m radii models

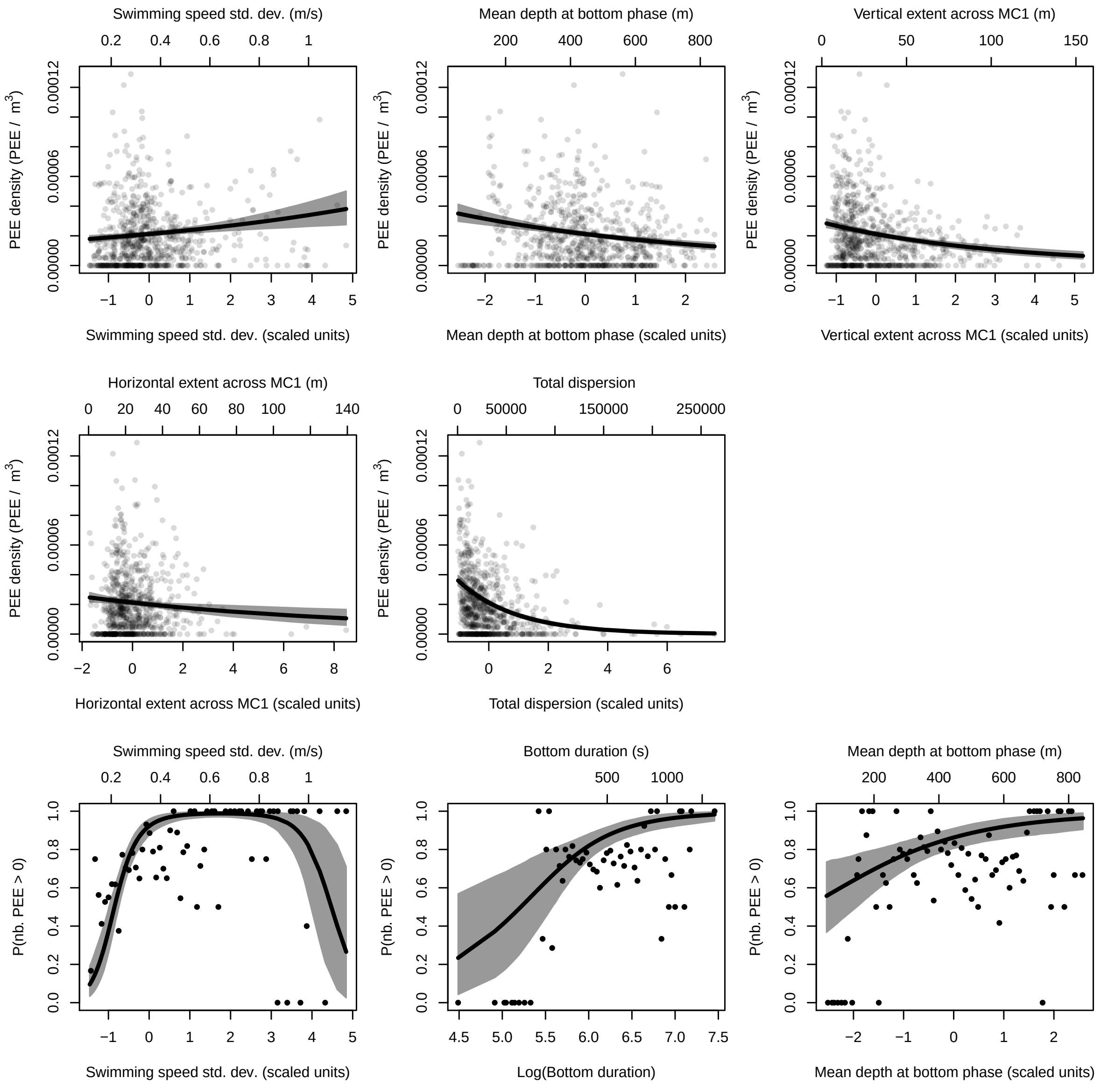

Figure 1: **Estimated relationships between the PEE density proxy and the descriptive parameters of the bottom phase trajectories. Results obtained for** 9 m **radius.** The top five graphics present the estimated effect of the count model and the bottom three graphics the estimated effects of the zero excess model. The tick black curves display the expected means at population level and the grey shades surrounding them stand for the 95% confidence interval of the expected mean.

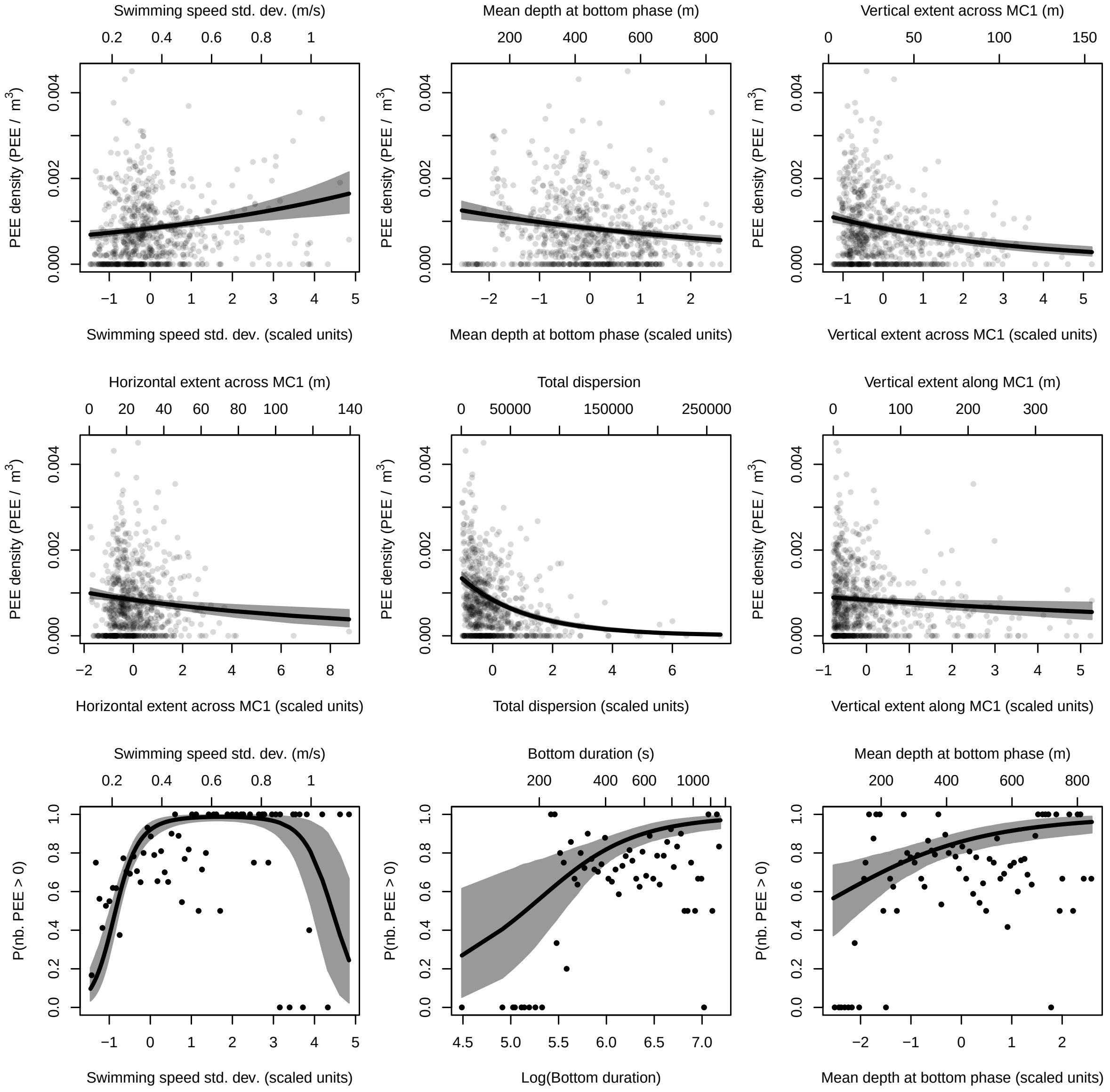

Figure 2: **Estimated relationships between the PEE density proxy and the descriptive parameters of the bottom phase trajectories. Results obtained for** 1.5 m **radius.** The top six graphics present the estimated effect of the count model and the bottom three graphics the estimated effects of the zero excess model. The tick black curves display the expected means at population level and the grey shades surrounding them stand for the 95% confidence interval of the expected mean.
